# Behavior of mice aboard the International Space Station

**DOI:** 10.1038/s41598-019-40789-y

**Published:** 2019-04-11

**Authors:** April E. Ronca, Eric L. Moyer, Yuli Talyansky, Moniece Lowe, Shreejit Padmanabhan, Sungshin Choi, Cynthia Gong, Samuel M. Cadena, Louis Stodieck, Ruth K. Globus

**Affiliations:** 1 NASA Ames Research Center, Space Biosciences Division, Moffett Field, CA 94035 USA; 2 Wake Forest School of Medicine, Obstetrics and Gynecology, Winston-Salem, NC 27101 USA; 3 Blue Marble Space Institute of Science, Seattle, WA 98154 USA; 4 Universities Space Research Association, NASA Ames Research Center, Moffett Field, CA 94035 USA; 5 San Jose State University, San Jose, CA 95192 USA; 6 KBRwyle, NASA Ames Research Center, Moffett Field, CA 94035 USA; 7 Novartis Institutes for Biomedical Research, Cambridge, MA 02139 USA; 8 BioServe Space Technologies, Department of Aerospace Engineering Sciences, University of Colorado, Boulder, CO 80302 USA; 9 Present Address: Utrecht University Graduate School of Life Sciences, Regenerative Medicine and Technology Program, Universiteitsweg 98, 3584 CG UTRECHT, The Netherlands; 10 Present Address: Keck School of Medicine of the University of Southern California, Department of Molecular Microbiology and Immunology, 2011 Zonal Avenue, Los Angeles, CA 90033 USA; 11 Present Address: Duke Empirical Inc., 2829 Mission St, Santa Cruz, CA 95060 USA

**Keywords:** Sensorimotor processing, Translational research

## Abstract

Interest in space habitation has grown dramatically with planning underway for the first human transit to Mars. Despite a robust history of domestic and international spaceflight research, understanding behavioral adaptation to the space environment for extended durations is scant. Here we report the first detailed behavioral analysis of mice flown in the NASA Rodent Habitat on the International Space Station (ISS). Following 4-day transit from Earth to ISS, video images were acquired on orbit from 16- and 32-week-old female mice. Spaceflown mice engaged in a full range of species-typical behaviors. Physical activity was greater in younger flight mice as compared to identically-housed ground controls, and followed the circadian cycle. Within 9–11 days after launch, younger (but not older), mice began to exhibit distinctive circling or ‘race-tracking’ behavior that evolved into a coordinated group activity. Organized group circling behavior unique to spaceflight may represent stereotyped motor behavior, rewarding effects of physical exercise, or vestibular sensation produced via self-motion. Affording mice the opportunity to grab and run in the RH resembles physical activities that the crew participate in routinely. Our approach yields a useful analog for better understanding human responses to spaceflight, providing the opportunity to assess how physical movement influences responses to microgravity.

## Introduction

In its 2011 report on the future of space exploration the National Research Council emphasized the importance of expanding NASA biosciences research to include long duration studies of rodents on orbit^1^. Spaceflight involves exposure to microgravity, radiation, isolation, confinement, increased CO2, and other factors that may culminate in stress and pose potential health risks for astronauts. Exposure to microgravity is associated with numerous physiological and neural alterations, including cardiovascular deconditioning, bone and muscle loss, immune and metabolic alterations, visual/ophthalmic changes, sensorimotor disturbances during acclimation and re-adaptation to 1 g, and decreased post-flight tolerance to orthostatic challenges^2^. The NRC recognized that that animal studies are needed to establish a deeper understanding of molecular, cellular, and organismal responses that can inform crew health during lengthy spaceflight missions. NASA Ames Research Center (ARC) responded to this charge by developing flight housing, operations, and science capabilities to support long duration rodent studies on the International Space Station (ISS).

Here we present a detailed behavioral analysis of mice living in space for extended durations (up to 37 days) as part of the NASA Rodent Research-1 (RR1) mission, the first deployment of the NASA Rodent Habitat on the ISS. Behavior comprises a remarkably well-integrated representation of the biology of the whole animal that informs overall neural and physiological status. Behavior not only ensures survival and reproductive success, it also provides a means by which the animal can control its environment to promote homeostasis^3^. Behavioral analysis can reveal how animals acclimate to the space environment, and how altered physical activity, feeding, drinking, circadian shifts, social interactions may alter other experimental measures. Without knowing what an animal is doing in space, it can be difficult to interpret findings acquired from a single organ, tissue, cell type or signaling pathway.

Cage environment also exerts major effects on rodent physiology and behavior^3,4^. These concerns are magnified in the weightless space environment where an animal’s interactions with its habitat and conspecifics are dramatically altered compared to 1 g conditions. Physical activity, shaped by habitat configuration and complexity, can affect a broad range of physiological parameters - heart rate, respiration, oxygenation, and blood flow among others - that may in turn affect morphology, biochemistry, gene expression, or other physiological measures derived from body tissues.

NASA Space Shuttle experiments have shown that, in microgravity, animal habitat configuration can alter physical activity, thereby resulting in differential effects on muscle morphology. Singly-housed rats flown in the smooth-walled Research Animal Housing Facility (RAHF) floated freely. In contrast, group-housed rats flown in the more complex Animal Enclosure Module (AEM) were in continuous physical contact with one another and with the habitat walls, actively twisting and turning in a sort of “biokinetic jungle gym”^5^. These results suggest that translating spaceflight research findings from model systems to humans involves distinguishing ‘direct’ effects of gravitational unloading on the body from ‘indirect’ effects due to behavioral alterations in weightless combined with animal habitat configuration. For all of these reasons, behavioral analysis of animals on-orbit is important for interpreting spaceflight data and ensuring translational relevance to human health in space.

Despite the obvious value of analyzing the behavior of model organisms *on orbit*, past spaceflight studies have typically flown animals in ‘black box’ environments that are ill-equipped to reveal what the animals are actually doing in-flight and how well they acclimate to living in space. Only a handful of reports have described the behavior of rodents during spaceflight. Two decades ago, adult, female (mid-pregnant) rats were flown five per NASA Animal Enclosure Modules (AEMs) on the Space Shuttle mid-deck (STS-66 and 72) for 11 and 9 days, respectively^6^. Brief video segments captured by the crew revealed a range of species-typical behavior, including eating, drinking, ambulation, self-grooming, and amicable social interactions.

In 2009, the Italian Space Agency flew the Mice Drawer System (MDS) on the ISS for 91 days, the longest duration rodent experiment ever conducted in space^7^. Three wildtype (wt) and three Pleiotrophin (PTN) transgenic mice that overexpress PTN under control of a human bone-specific osteocalcin promoter were housed individually. Exploration, self-grooming, sniffing, resting, eating and drinking, floating, hanging by the forelimbs, ambulating across grid bars, and contact with an object presented into the cage were reported. Only half of the mice survived.

In 2013, 45 male mice were flown for 30 days on the unmanned Bion-M1 mission in mice housed three per Russian Block Obespecheniya Soderzhaniya (BOS) (“Unit for the Provision of Housing”) habitat. A food dispenser malfunction resulted in approximately 50 percent survival^8^. In-flight video segments revealed higher levels of aggregative behavior (huddling contact) near the feeder relative to identically housed ground controls. Unfortunately, camera visibility declined precipitously after the first flight week, significantly compromising analysis of the vast collection of video segments acquired on orbit.

Qualitative behavioral observations of individually housed mice flown for 35 days in Japan Aerospace Exploration Agency (JAXA) Habitat Cage Units (HCUs) revealed that mice floated freely throughout the habitat, utilizing their tails to maintain their posture while resting^9^. Collectively, these reports are intriguing, but they demonstrate the paucity of rigorous behavioral analysis in studies of spaceflown rodents.

**Figure 1:**
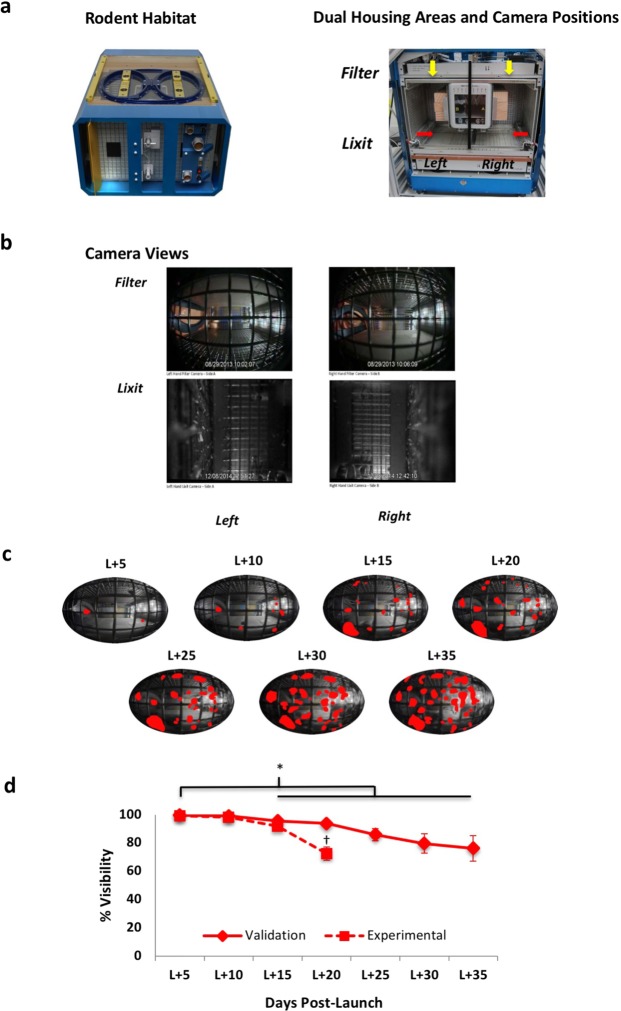
Rodent habitat (RH), camera views and field visibility. (**a**) Left. RH unit with two access ports on top. Right. Dual housing areas with two camera positions per compartment shown in 1 g orientation. Each RH is bisected by a grate (depicted by the black vertical line) yielding left and right caging compartments housing five mice per side. Within each compartment, one camera was mounted in close proximity to the waste filter (yellow arrows depict ‘Filter’ camera), and the other camera in close proximity to the Lixit tubes (red arrows depict ‘Lixit’ camera) mounted on the water reservoir. (**b**) Images acquired from corresponding left and right camera locations. (**c**) Digitized images captured at 5-day intervals beginning on Launch (L) + 5 (first full ISS mission flight day) derived from the right filter view (Validation cohort). Obscured areas colorized in red (Adobe Photoshop CC 2014) represent debris accumulation on the camera lens. Binary images were created, then proportions of obscured versus non-obscured pixels calculated using Image J (http://rsb.info.nih.gov/ij/). (**d**) Percent (mean +/− se) in visibility over time was calculated using Image J (http://rsb.info.nih.gov/ij/). On L + 20, the final mission day for Experimental mice, camera visibility for Validation and Experimental mice did not differ statistically from one another, although a trend toward poorer visibility of Experimental relative to Validation mice was observed. Photo Credits: NASA.

To gain greater insights into mouse behavior on orbit, we conducted a systematic and detailed analysis of species-typical behavior, physical activity, and circadian cyclicity in mice flown on the RR1 mission. RR1 was comprised of two distinct mouse cohorts: Validation mice were young (16-week at launch) female C57BL/6J mice flown by NASA to confirm biocompatibility of the RH during a long-duration (33-day) mission. Experimental mice were older (32-week at launch) female MuRF1 knockout (KO) mice and wild-type (wt) littermates (C57BL/6NTac background) were on ISS for 17–18 days. Bone, muscle and other physiological responses were analyzed by the Novartis Institutes for Biomedical Research (NIBR) in collaboration with the Center for the Advancement of Science in Space (CASIS). For each mouse cohort, ten mice were assigned assigned to Flight (FLT), identically-housed ground control (GC) or Vivarium (VIV) cage conditions. Video images were acquired from RH-housed FLT and GC mice only. Mice were housed in groups of five per each side of the bisected RH units (Fig. 1). There were two groups of Validation mice and one group each of MuRF1 KO and wt Experimental mice in the study.

The RR1 video collections comprise an extensive and rich repository of images for analysis. However Video image acquisition was planned to conduct daily health checks, rather than detailed behavioral analysis. As a result, it was not possible to analyze behavior in the cage area adjacent to the water reservoir, there was limited tracking of individual mice due to the absence of visible mouse identifiers, and sample durations varied across mission days with no light cycle footage for much of the mission.

The Supplementary Video shows images of the mices’ behavior on orbit. Our major findings are: (1) FLT mice engaged in similar forms and levels of various species-typical behaviors as compared to GC mice, and (2) emergence of a unique circling or ‘race-tracking’ behavior within two weeks of launch in younger (but not older) mice. Circling swiftly metamorphosed into a coordinated group activity. Stereotyped motor behavior, rewarding effects of physical exercise, and vestibular sensation evoked via self-motion are considered as viable explanations for circling on orbit.

## Results

### Mission outcome

All 20 Validation and Experimental spaceflight mice survived their respective mission durations. The mice showed consistent, robust levels of physical activity throughout the mission. Post-flight, body weights were comparable to GC mice, and coat condition was excellent indicating that grooming was effective^10^.

### Video image quality

Past rodent spaceflight studies^6,8^ have reported substantial degradation of Video images due to the accumulation of debris and floating liquids on camera lenses. We first determined whether the RR1 Video image resolution was sufficient for behavioral analysis by quantifying lens visibility in each of the bisected Validation and Experimental RH units across flight days for both flight and ground views (Fig. 1c,d). Approximately two weeks into the flight, visibility of images captured from each of the four flight housing areas showed a significant but modest decline that persisted throughout the mission, reaching a maximum difference from L + 5 on the final video acquisition day. For Validation groups, final video acquisition day (L + 35) visibility was 85 and 67 percent for the left and right views, respectively. Experimental group visibility on the earlier final video acquisition day (L + 20) was 67 and 77 percent for the left and right views. Since these groups were flown in the same RH hardware, and the Experimental mice were twice the age of the Validation mice, the 32 percent greater biomass of the Experimental relative to Validation Flt mice (Pre-Launch body mass (g) Mean +/− SD, Experimental, 28.8 +/− 2.0; Validation, 19.5 +/− 1.1) likely accounts for the approximately two times more rapid decline to a lower final percent visibility relative to Validation mice. Collectively, this analysis reveals that the RH habitat design and Filter area video configuration provide excellent visibility and quality Video images that are amenable to highly detailed behavioral analysis for extended missions.

### Behavior of validation mice

#### Species-typical behavior

**Figure 2:**
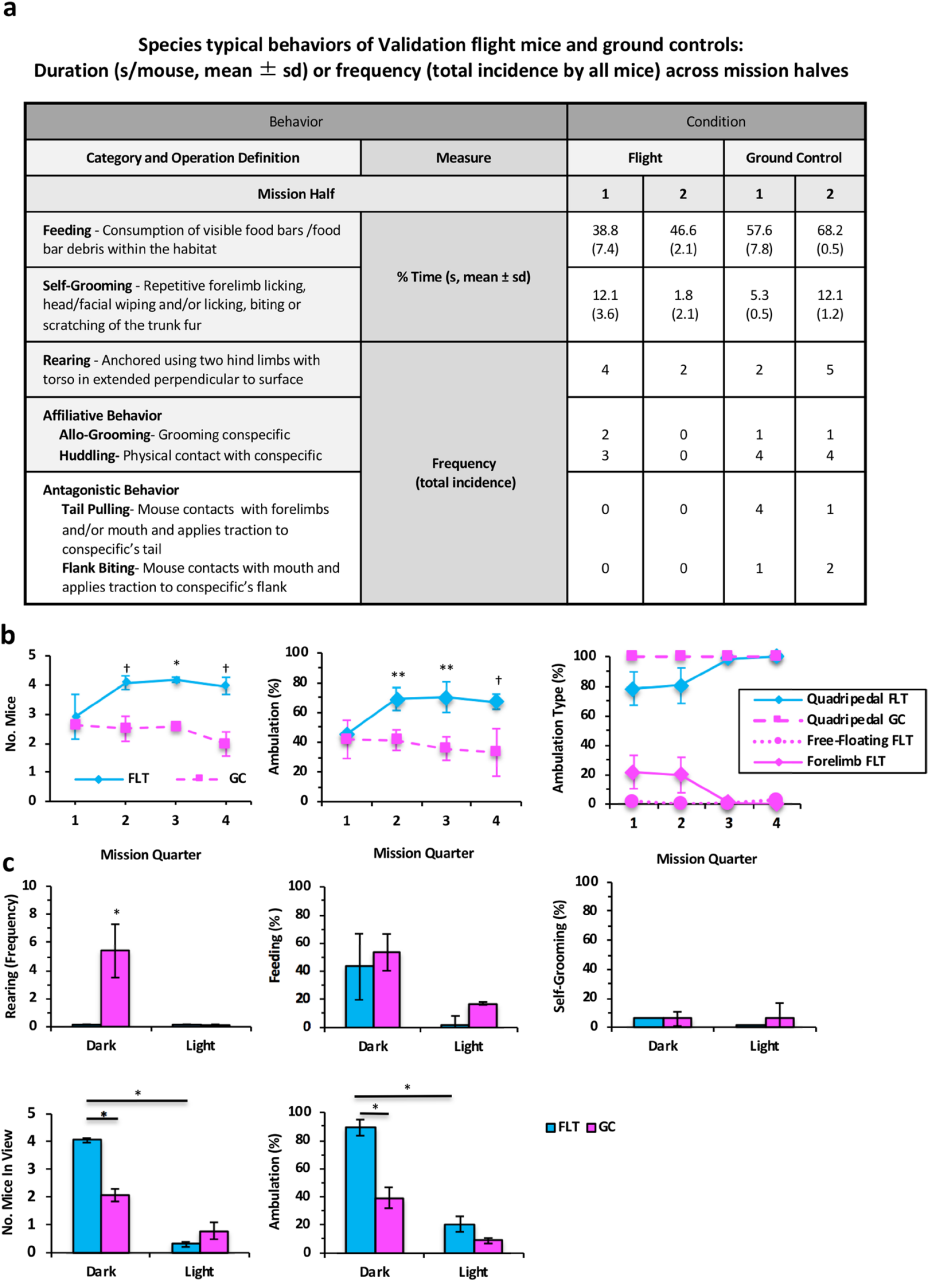
Behavioral of Validation mice on orbit and ground controls. (**a**) Duration (s/mouse, mean +/− sd) weighted for daily sample duration and number of mice (*100) engaged in the behavior or frequency (total incidence) of discrete categories of species typical behavior by condition during dark cycle epochs across mission halves for Validation mice. (**b**) Validation Flight (FLT) and Ground Control (GC) mouse presence and ambulatory behavior within the Filter area of the Rodent Habitat (RH) scored during the dark cycle on each mission day averaged (mean +/− se) across 8-day ISS mission quarters. Left. Average (mean +/− se) number of FLT and GC mice in Filter view. Center. Percent time ambulating by FLT and GC mice during the dark cycle in the Filter area across mission quarters. Right. Percent time (across mission quarters) that FLT mice engaged in quadrupedal ambulation, forelimb ambulation, or utilized no limbs while moving (free-floating) compared to GC mice. (**c**) Around-the-clock video surveillance of NASA FLT and GC mice hourly across light and dark cycle phases on L + 30 (mean +/− se) normalized to total video duration and numbers of mice. Upper images. Rearing frequency of FLT and GC mice (left). Number of Validation FLT and GC mice feeding (middle) and self-grooming (right). Lower images. Number of FLT and GC mice in view during the dark as compared to light phase of the cycle (left). Number of FLT and GC mice ambulating during the dark cycle phase (right).

Quantitative analysis of behavioral categories supported our prediction that spaceflight and control mice would engage in similar types and levels of species-typical behaviors (Fig. 2a). To account for measures from multiple individuals within the group over varying sample durations, the total amount of each species-typical behavior observed in all mice was scored, normalized to video sample duration for the corresponding flight day, and adjusted to an average *per mouse* values. Data are presented as either duration (s) of time or frequency that mice are engaged in a particular behavior. Due to the absence of visible identifying markers, mouse group (left versus right habitat side), rather than individual mouse, was considered to be the unit of statistical analysis.

Feeding and self-grooming were observed throughout the mission, with consistent levels of occurrence across microgravity and 1 g conditions. Drinking behavior could not be quantified due to water access in the Lixit area only. Hindlimb rearing, a characteristic species-typical exploratory behavior in rodents typically defined in terrestrial studies as lifting of the forebody from a quadrupedal stance against the gravity vector, was observed in control mice. Notably, spaceflight mice were observed to tether themselves to the habitat walls with their hindlimbs and/or tails, and extend their forebodies similar in form to hindlimb rearing.

Some characteristic mouse behaviors may have occurred more commonly within the Lixit area, where they could not be readily quantified due to the limited camera FOV. Social interactions, both amicable (i.e., huddling, allo-grooming) and antagonistic (i.e. tail pulling, biting), all of which are common mouse behaviors, were observed infrequently in both flight and control conditions. These behaviors may also have occurred in the Lixit area that was approximately 20 percent smaller volume than the Filter area. This would afford more frequent opportunities for physical contact between conspecifics.

#### Numbers of mice in view

Numbers of FLT mice in view (i.e., present in the Filter) compared to GC mice increased after the first mission quarter (Fig. 2b). Similar numbers of Validation FLT and GC mice were observed in view during the dark phase of the cycle during Q1 (ns). In Q2, FLT mouse presence in the Filter area appeared elevated in FLT relative to GC mice, but did not achieve statistical significance (p = 0.085; ns). During Q3, an increase in FLT mouse presence in the Filter area achieved significance in Q3 (p = 0.006), but did not emerge in Q4 (p = 0.059, ns). (Alpha = 0.0125).

#### Ambulation

Ambulation was defined as mouse movement from place-to-place, distinct from movements performed while stationary such as grooming and feeding. Percent time spent ambulating and type of movement (quadrupedal, forelimb, or free-floating) were quantified from a single mouse for two minutes each day (Fig. 2b). During Q1, FLT and GC mice spent similar amounts of time (40–45 percent) moving. While GC mice ambulated 35–40 percent of the time during Q2–4, FLT mice exceeded 60 percent time during the same observation period (ns; Q2, p = 0.058; Q3, p = 0.058; Q4 = 0.09).

We analyzed the morphology of ambulatory behavior, comparing quadrupedal movements (active use of all four limbs), forelimb movements (active use of both forelimbs), and free-floating (movements with no limb involvement). Free-floating was observed in spaceflight video segments but surprisingly never exceeded 3 percent of the total physical activity. Spaceflight mice (FLT) initially engaged in forelimb ambulation, movement style never observed in 1 g controls. Forelimb ambulation was observed in FLT mice 21 percent of the time during the first half of the mission but <1 percent thereafter. Overall, far less forelimb as compared to quadrupedal was observed in FLT mice over mission quarters. Importantly, spaceflight mice discontinued forelimb ambulation in favor of quadrupedal ambulation during the second half of the mission, providing evidence that spaceflight mice adapted their style of ambulation as they acclimated to the weightless space environment.

We analyzed the morphology of ambulatory behavior, comparing quadrupedal movements (active use of all four limbs), forelimb movements (active use of both forelimbs), and free-floating (movements with no limb involvement). Free-floating was observed in spaceflight video segments but surprisingly never exceeded 3 percent of the total physical activity. Spaceflight mice (FLT) initially engaged in forelimb ambulation, movement style never observed in 1 g controls. Forelimb ambulation was observed in FLT mice 21 percent of the time during the first half of the mission but <1 percent thereafter. Overall, far less forelimb as compared to quadrupedal was observed in FLT mice over mission quarters. Importantly, spaceflight mice discontinued forelimb movements in favor of quadrupedal movements during the second half of the mission, providing evidence that spaceflight mice adapted their style of ambulation over time as they acclimated to the weightlessness space environment.

#### Circadian analysis

On L + 30, around-the-clock hourly video sampling was performed. Rearing frequency (Fig. 2c) was observed in GC mice only during the dark cycle (p = 0.049). Feeding and self-grooming were not increased in either treatment group during the dark cycle. Hourly around-the-clock comparisons revealed a significant increase in the numbers of FLT mice in the Filter view during the dark cycle as compared to GC mice (p = 0.0009). FLT mice also spent significantly more time in the Filter area of the habitat during the dark as compared to light cycle (p = 0.011), whereas this metric did not achieve significance for GC mice (ns). As compared to GC mice, time spent ambulating was significantly elevated in FLT mice during the dark cycle (p = 0.05), and both FLT and GC mice were significantly more active during the dark as compared to light cycle (FLT, p = 0.01; GC, p = 0.04). This analysis indicates that the circadian timing system was intact during the last mission quarter. Total time FLT mice spend ambulating on L + 30 was more than double that of GC mice during the dark cycle. These data are accounted for, in part, by spontaneous emergence and persistent occurrence of circling behavior in spaceflown Validation mice. (Alpha level = 0.025).

#### Circling

**Figure 3:**
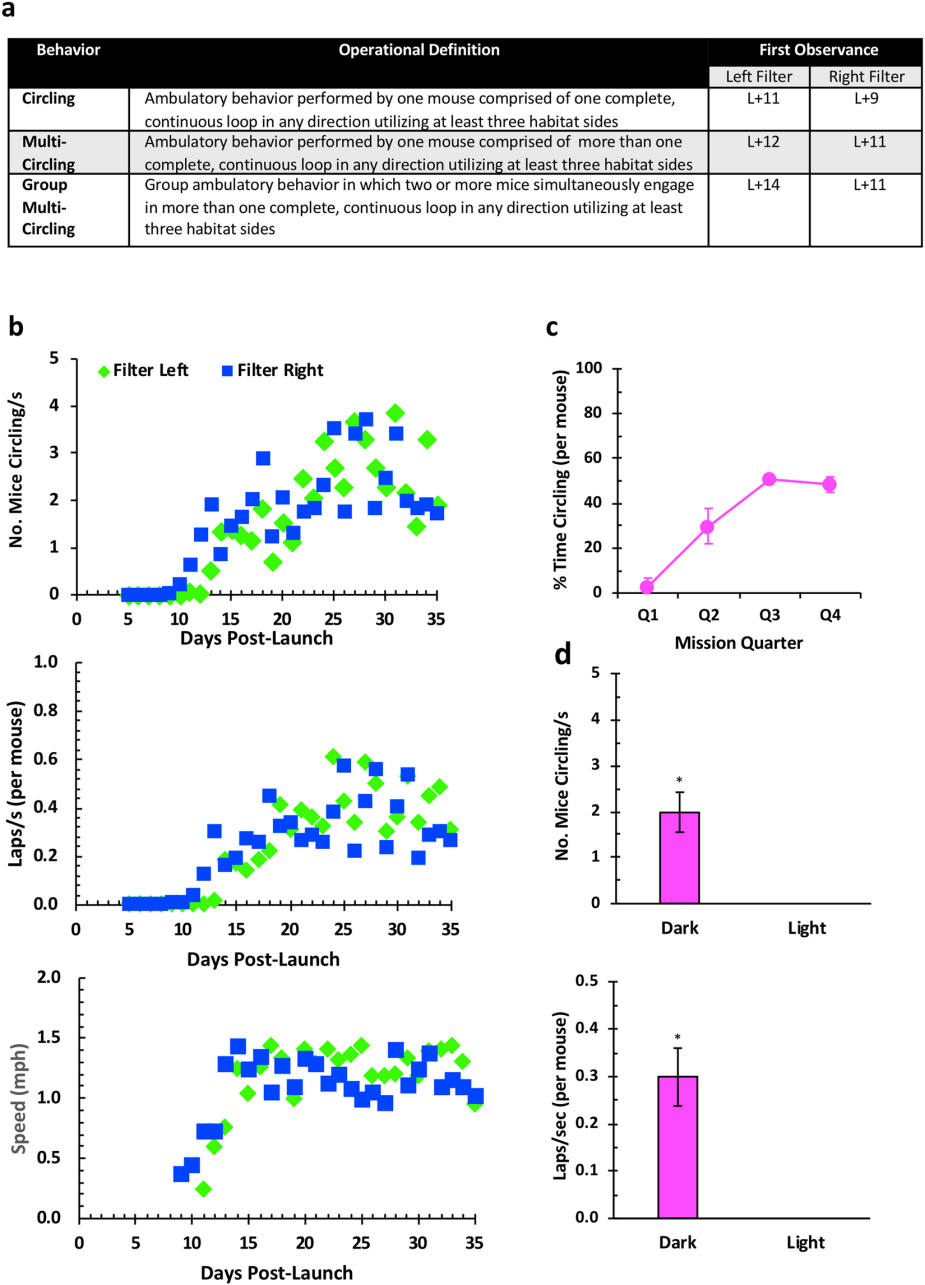
Circling in Validation mice on orbit. (**a**) Table 2 Sequence of milestone appearance and operational definitions in the emergence of circling behavior. (**b**) Quantification of circling behavior. Top image. Average number of mice circling by cohort (Filter Left or Filter Right) normalized to total video duration. Middle image. Average number of laps per second per mouse by cohort (Filter Left or Filter Right) normalized to total video duration. Bottom image. Estimated average circling speed (m/s) [Formula: Estimated lap distance traveled assuming route is an average between an oval and a rectangle/number of video frames traversed in one lap * frame rate (29.97 fps)] was converted to mph (m/s * 2.2369). (**c**) Percent time spent circling (per each of the five mice within each of the two Validation FLT groups) averaged across 8-day mission quarters. (**d**) Around-the-clock analysis of circling behavior in Validation mice. Top image. Average (mean +/− se) number of mice circling per second averaged across light and dark cycle phases derived from video segments acquired hourly beginning on L + 30. Bottom image. Average (mean +/− se) laps per second normalized for numbers of Validation mice circling.

Within 8–10 days post-launch (3–5 days post-ISS docking), the Validation FLT mice began to engage in a unique circling or ‘race-tracking’ behavior in which they moved their bodies along an ovular trajectory within the Filter area of the RH utilizing the habitat walls (See Supplemental Video). Circling emerged spontaneously, following an organized progression beginning with back-and-forth running by mice along habitat surfaces, and propelling themselves by pushing off of walls with hindlimbs. This behavior quickly evolved into full circular laps (Fig. 3a). Once mice began to navigate themselves in a complete lap within the habitat (L + 8 and 10 for the left and right sides of the habitat, respectively), the behavior progressed within 1–2 days to consecutive laps (Multi-Circling). Coordinated group circling (Group Multi-Circling) emerged 1–2 days later and involved multiple mice circling at the same time. The entire progression from individual single circles to group multi-circling behavior developed in both NASA Validation cohorts over just three days. Periodically across the flight, mice displayed backward flipping or “somersaulting” behavior similar to stereotypic mouse behavior described in terrestrial laboratories^3^. Notably, Validation GC mice and Experimental FLT and GC mice did not somersault.

Circling participation and lap rate (Fig. 3b) began abruptly, then gradually increased to high levels maintained over the remainder of the mission. Once circling emerged as a predominant activity, average speed remained consistent spanning approximately 1.08 and 1.39 mph (0.48–0.62 m/s). Using a one-sample t-test comparing to a constant of zero, L + 12 was the first day that speed significantly differed (p = 0.004), and significance was maintained for each subsequent day of the study. Circling participation, lap rate, and average speed showed similar patterns for mice housed in each side of the Validation mouse habitat.

Percent time spent circling (adjusted for numbers of mice) across the four mission quarters (Fig. 3c) reflected the observation that circling began to emerge during Q2 (p = 0.07, ns), achieving statistical significance during Q3 (p = 0.005), and remained elevated during Q4 but did not achieve significant (p = 0.036). (Alpha = 0.0125). Twenty-four-hour analysis beginning on L + 30 revealed the presence of circling behavior during the dark phase of the cycle only (Fig. 3d; Percent time circling per mouse, p = 0.012, and average number of laps per mouse, p = 0.013).

### Behavior of experimental versus validation mice

Table 1 compares major behavioral categories and ambulation/activity measures for the Validation/Experimental and FLT/GC conditions across the first two flight quarters. Compared to the Experimental group, the Validation group showed increased numbers of GC mice in view during Q1, increased numbers of FLT mice in view during Q2, and decreased feeding in FLT mice during Q1. FLT/GC comparisons revealed increased feeding and grooming in Experimental FLT mice during Q1, and increased circling in Validation FLT mice during Q2. Experimental mice exhibited a significant decline in grooming in Q2 as compared to Q1.

**Table 1:** Dark Cycle Phase Behaviors (mean and range) in Validation (16-week-old) and Experimental (32-week-old) Flight and Ground Control Mice Across RR1 the First and Second Mission Quarters.

Condition	Ground				Flight			
Group Behavior	Validation (range)		Experimental (range)		Validation (range)		Experimental (range)	
Mission Quarter	Q1	Q2	Q1	Q2	Q1	Q2	Q1	Q2
No. Mice In View	2.62^a^ (2.58–2.66)	2.49 (2.06–2.93)	1.51^a^ (1.40–1.63)	2.01 (2.01–2.01)	2.91 (2.14–3.68)	4.08^b^ (3.84–4.31	2.8^h^ (2.55–3.06)	2.29^b,^^h^ (2.06–2.51)
% Feeding	58.97 (52.10–65.83)	71.22 (63.15–79.29)	41.33^*e*^ (38.98–43.69)	76.57 (50.55–102.59)	33.60^*c*^ (27.50–39.70)	44.08 (39.14–49.02)	77.72^*c,e*^ (76.32–79.12)	85.70 (66.13–105.27)
% Grooming	4.86 (3.52–6.21)	2.13 (0.11–4.14)	2.52^f^ (1.10–3.95)	5.56 (1.98–9.13)	20.93 (15.00–26.88)	6.36 (1.43–11.31)	13.84^f,l^ (13.20–14.48)	4.14^l^ (3.09–5.20)
% Total Time Ambulating	41.84 (28.97–54.70)	41.32 (34.38–76.62)	38.00 (29.70–46.30)	50.13 (43.25–57.00)	45.38 (44.78–45.97)	55.50 (34.38–76.62)	14.67 (6.70–22.70)	45.13 (43.25–57.00)
% Quadrupedal Ambulation	100 (100–100)	100 (100–100)	100 (100–100)	100 (100–100)	76.89 (65.26–88.54)	78.58 (63.66–93.50)	99.44 (98.90–100.00)	94.29 (90.9–97.7)
% Forelimb Ambulation	0 (0–0)	0 (0–0)	0 (0–0)	0 (0–0)	21.52 (9.02–33.99)	18.93 (6.50–31.35)	0 (0–0)	5.13 (1.20–9.10)
% Free Floating	0 (0–0)	0 (0–0)	0 (0–0)	0 (0–0)	1.59 (0.74–2.43)	2.49 (0.00–4.99)	0.56 (0.00–1.1)	0.58 (0.00–1.2)
Time Circling (% mouse)	0 (0–0)	0^g^ (0–0)	0 (0–0)	0 (0–0)	2.91 (0.24–5.57)	29.86^d,g^ (24.28–35.44)	0 (0–0)	0.29^d^ (0.00–0.29)

Legend: p-values corresponding to 2-tail t-tests (unless otherwise indicated).

Validation vs Experimental: significant, ^a^0.012 (alpha = 0.017); trend for: ^b^0.032; ^c^0.02; ^d^0.033 (alpha = 0.033).

Flight vs Ground Control: significant, ^e^0.006 (alpha = 0.017); trend for: ^f^0.019, ^g^0.028 (alpha = 0.033).

Q1 vs Q2: trend for: ^h^0.027 (alpha = 0.033).

Circling was nearly absent in Experimental mice. MuRF1 KO, but not wt mice, displayed circling but at an average of < 1 percent of video duration (a total of 21 laps) compared to 24 percent (a total of 3,267 laps) for Validation mice during the same time period. This was true despite approximately ten times more video footage (longer daily video segments) for Experimental animals across ISS days 5–20.

## Discussion

All twenty mice flown on the first foray of the NASA Rodent Habitat on ISS maintained excellent health during the mission. Qualitative observations indicated that spaceflight mice readily adapted to the RH, propelling their bodies freely and actively throughout the habitat, utilizing the entire volume of space available to them. Over time, the spaceflight mice began to move more quickly throughout the habitat, translating with ease through open spaces, but also anchoring their bodies using tails and/or paws. Anchoring allowed mice to feed, self-groom, huddle, and engage in social interactions. Mice remained active and mobile throughout the experiment, exploring their environment and occupying all areas of the habitat. The unique circling behavior emerged during the second mission quarter and progressed from a relatively solitary behavior to a highly coordinated group activity.

Here we report that spaceflight mice preferred the spacious Filter area over the smaller Lixit area of the habitat. This was evidenced by increased numbers of flight versus control animals in the Filter view beginning during the second mission week. With its larger expanse, the cage volume captured by the Filter camera provides greater opportunities for mobility relative to the Lixit area, and is therefore uniquely permissive for circling behavior. Further, circling emerged at the same time that mice increased their presence in the Filter area of the cage, raising the possibility that spaceflight mice developed a preference in flight for the larger cage area where circling could be performed. It is noteworthy that circling has been observed in at least one past shuttle mission^11^ utilizing the Animal Enclosure Module (AEM), a precursor to the RH with considerable design overlap. This behavior has also been reported in mice flown on ISS in the RH on missions following RR1^10^.

Studies of mice flown in habitats other than the AEM or RH have not reported either circling or increased levels of physical activity or ambulation during spaceflight relative to 1 g controls^7–9^. This could be related to the design of the RH, that in contrast to some other habitats, is configured with multiple surfaces that mice can grab and utilize that may facilitate ambulation throughout the habitat. However there could be other explanations for the circling behavior. In the present study, circling by older Experimental mice was less than 1 percent of the numbers of circling laps observed in younger Validation mice. Age-related decline in physical activity in laboratory rodents, even across the range of young to middle-age, is well-established^12^ and may have been a determinant of circling behavior in 16- but not 32-week-old mice reported here. This interpretation is supported by reports on subsequent RR flights of circling behavior in younger mice acquired from both Jackson Laboratories and Taconic Biosciences suggesting that age plays a role in the emergence of circling rather than strain differences. While additional experiments are clearly needed to ascertain the precise factors underlying mouse circling on orbit, here we consider several possible explanations.

Circling could represent the emergence of stereotyped motor behavior or abnormal repetitive behaviors (ARBs). Repetition comprises an important feature of normal behavioral functioning across animal phyla^13^, however stereotypic behaviors are repetitive, unvarying, and apparently functionless behavior patterns^3,14^. Stereotypies are generally thought to reflect impaired welfare as they tend to spontaneously appear in barren or restricted housing conditions^3,14–16^. Common stereotypies^3,14,16^ that have been observed include pacing in birds, prosimians and large carnivores, crib- and bar-biting in horses, pigs and mice, vertical jumping in mice, rocking in primates, and self-injurious behaviors in parrots and primates.

Although less common in mice, “somersaulting”, “route-tracing”, and other forms of repetitive, unvarying and functionless locomotor have been observed^3^. Indeed, circling exhibited by Validation spaceflight mice shares some common characteristics with stereotypic behavior, viz., highly repetitive, somewhat invariant, with no obvious goal. In addition to the rapid, smooth circling trajectories, mice occasionally exhibited rapid back-flipping, not unlike somersaulting reported in the mouse husbandry literature. Free-fall is stressful and can activate immune responses^17^, and stereotypies can be triggered or intensified by emotional stress^18^. Notably, ground control mice housed in the RH did not exhibit somersaulting or any other identifiable motor behavior. This is similar to looping or somersaulting reported in tadpoles, fish and birds exposed to microgravity during parabolic or spaceflights^19^. These observations indicate that mouse circling in flight was not due to a housing effect alone but raises the distinct possibility of an interactive influence of housing and weightlessness.

Environment enrichment is a biologically relevant resource or structuring of the cage that facilitates the occurrence of highly motivated natural behavior^20^. As such, enrichment can successfully reduce or prevent the occurrence of maladaptive behaviors such as ARBs^3^, and improves translation animal studies to humans^4^. For mice, bedding is considered a highly effective form of environment enrichment that enables species-typical foraging and nest-building behaviors, and promotes warmth^15^. An animal is likely to be under a state of stress when its ability to perform natural, species-typical behaviors, such as foraging or nest-building, are prevented^3,20,21^. In this study, the RH was not configured with environmental enrichment typical in terrestrial laboratories. However identically-housed ground controls did not exhibit analogous behavior, therefore a lack of enrichment and/or foraging opportunities alone does not support the interpretation of circling as a motor stereotypy. Microgravity was clearly a necessary condition for the emergence of circling. Notably, mice prefer a three-dimensional, complex cage structure that facilitates climbing and locomotion^20^. The ability of mice to utilize the full volume of the RH under microgravity conditions could, in and of itself, serve as an effective enrichment.

The mices’ circling behavior has some similarities to their use of running wheels in terrestrial studies. Wheel running is considered to be a paramount form of enrichment for rodents, one that occurs at far greater rates in barren cages as compared to enriched ones^22,23^ or under stressful conditions^23–25^. Mice given the opportunity to use a running wheel in the wild will do so with bout lengths comparable to captive mice^26^ suggesting that running wheel activity in the laboratory setting is an elective behavior. It has been argued, however, that voluntary wheel running in the wild is not sufficient to preclude the possibility that wheel running in the laboratory itself represents a motor stereotypy^21^ with the potential to become compulsive and self-reinforcing^27,28^.

Terrestrial laboratory animals housed in colony environments often experience environmental stress^29^. It’s likely that hypergravity during launch, and microgravity (free-fall), weightlessness, increased airflow, low ambient temperature, increased CO2 and other environmental changes in space are stressful. Circling behavior in this study could have emerged as a stress response, however it would need to be argued spaceflight was uniquely stressful for the Validation mice that were younger in age and acquired from the Jackson Labs as compared to older Experimental mice acquired from Taconic Biosciences. Importantly, none of the Validation mice showed overt physiological signs of chronic stress or compromised health or welfare raising doubt that spaceflight mice circled due to stress.

For example, amounts of time spent feeding, and post-flight body weights were comparable in flight and ground control mice in both age groups. Antagonistic behaviors were observed at low levels in all study groups. Post-flight examination of the carcasses preserved on-orbit confirmed excellent coat condition with no evidence of ‘barbering’, a mouse obsessive/compulsive behavior involving abnormal whisker- and/or fur-plucking that is homologous to trichotillomania, human compulsive hair pulling^30^.

There is increasing recognition that physical exercise exerts positive, rewarding effects on brain and behavioral health, and can help combat anxiety, depression and cognitive impairment^31,32^. Voluntary running in young adult mice reduces depressive and anxiety-like behavior^33–35^. Stress-protective effects of exercise arising from changes to neural systems include enhanced galanin-mediated suppression of brain norepinephrine^36,37^ and cortical regulation of polyunsaturated fatty acids (PFAs)^38,39^ that exert positive effects on mood^40,41^.

Voluntary wheel running has been shown to be rewarding and to activate brain reward pathways with effects on the brain mimicking those induced by natural rewards and drugs of abuse^42,43^. The dopamine system (viz., D2 and D3 receptor activation) involved in movement, and to a lesser extent, in enhanced corticosterone synthesis, appear to contribute to exercise motivation^44^. Physical exercise, including locomotor activity in mice and rats, and flying in birds, exerts significant influence(s) on growth factors and neuropeptides leading to structural changes in the brain^45–47^ including enhanced hippocampal neurogenesis and angiogenesis, and substantial increases in gray and white matter volume in multiple cortical area and hippocampus^45–47^. Circling behavior may relate to one or more potentially rewarding or anxiety-reducing changes in rodent brains. Future research comparing brain morphological, physiological and biochemical changes in mice that circle in space is warranted.

Microgravity exposure eliminates vestibular sensory input to the otolith organs (i.e. the utricle and saccule) that detect and respond to head static position and linear acceleration^48^. The otoliths also contribute to the perception of verticality and spatial navigation in the neocortex and limbic system, including the hippocampus^47^. Changes in vestibular reflexive function and perception commonly occur during spaceflight Ground-based studies of rodents in which Bilateral Vestibular Labrynthectomy (BVL) is performed (sur- gical, chemical, or genetic lesions induced) report post-operative emergence of persistent motor abnormalities including hyperactivity, circling, and moderate ataxia^49–51^. The activity of forebrain dopamine systems was thought to play a central role in motor abnormalities following BVL, however the findings have been inconsistent^51,52^ and vestibular loss-related motor disorders cannot be solely explained by dopaminergic (DA) alterations^53^. Interestingly, mice flown for 30-days on the Bion-M1 mission showed decreased expression of crucial genes involved in DA synthesis and degradation, as well as the D1 receptor^54^. Other studies have focused on the orexin/hypocretin system implicated in central motor control^55^, a role that is supported by findings that altering OXA expression may contribute to hyper-locomotion following an acute vestibular lesion^51^. Anxiety-like responses have also been reported following BVL^49,52^, raising the possibility that circling served as an anxiolytic to offset effects of otolith unloading.

Fish exposed to microgravity exhibit somersaulting and looping (swimming in tight circles) and spin- ning^56–58^. In a simple, but clever, experiment, Anken and colleagues analyzed Video images of fish looping behavior during a parabolic flight in relation to post-landing otolith weights^56^. The asymmetry in otolith weight was highly correlated with the direction and extent of the behavior looping. Thus microgravity reveals the natural asymmetry in neural structures that have been neutralized in gravity through adaptation.

An intriguing possibility is that, in the absence of gravitational input to the otoliths, circling behavior by the Validation spaceflight mice generated biologically-relevant amounts of vestibular sensory input. It has been hypothesized that stimulation of the vestibular system during self-motion could play a regulatory role in brain changes associated with physical activity^45^. The canals are functional in microgravity and presumed to be actively monitoring circling movements. It is reasonable to postulate that self-motion behaviors evoke vestibular stimulation in space.

The novel idea that natural, everyday activities generate measurable vestibular stimulation was recently examined in mice^59^ by recording mouse head movements using a lightweight module combining three linear accelerometers to measure linear accelerations, and three gyroscopes to measure angular accelerations during species-typical behaviors (e.g., walking, running, foraging, grooming, eating, climbing, etc). The intensity of the stimuli ranged from approximately 500 to 1300 deg s-1 angular velocity and 1–4.5 G linear acceleration providing evidence that the animals’ behavioral repertoire generates natural vestibular signals reaching the vestibular end organs. Thus it is feasible to suggest that self-motion could provide biologically-relevant amounts of stimulation to the vestibular end-organs during spaceflight. In the present study, we calculated from the RR1 in-flight Video images from L + 12 through L + 35 the average circling speed for the Validation mice. Assuming an ellipsoidal path around the habitat measuring an average of 27.2 inches in circumference, we estimated an average acceleration at a range of 1.16 m/s2 to 1.82 m/s2 (0.12 g-0.186 g). Vestibular self-stimulation poses a viable explanation for the circling behavior reported in this study. Spaceflight studies comparing mouse circling in different cage configurations (e.g., round vs oval) are needed to explain why mice are circling on orbit.

The behavioral analysis reported here provides a deeper understanding of how mammals acclimate to extended spaceflight, and sets the stage for identifying the physiological, cellular and molecular events driving mouse circling behavior in space. Stereotyped motor behavior, rewarding effects of physical exer- cise, and vestibular sensation produced via self-motion are controlled by distinct sensory-motor and brain areas for which specific, testable hypotheses can be generated. For example, signaling in stress pathways (hypothalamic-pituitary-adrenal axis; HPAA) of circling mice would suggest classification of circling behavior as a motor stereotypy whereas reward centers (primarily the cortico-basal ganglia-thalamo-cortical loop) would be involved if circling exerts a positive reinforcing effect. Vestibular self-stimulation would involve activity within the otolith organs and central nuclei of the balance system and may overlap with activation of stress and/or reward pathways. Behavioral research is vital for ensuring fidelity in translating rodent studies to human health concerns in space. Affording mice the opportunity to grab and run in the RH resembles physical activities that the crew participate in routinely. Our approach is yielding an interesting analogue for better understanding human responses to spaceflight, and providing the opportunity to begin to address how physical movement influences responses to microgravity.

## Methods

### Mice and spaceflight mission

 C57BL/6J Flight (FLT; N = 10) and Ground Control (GC; N = 10) Validation mice were obtained from The Jackson Laboratory (Bar Harbor, ME), were used for Validation studies. Ten female muscle RING finger protein 1 (MuRF1) KO mice randomized to FLT (N = 5) or GC (N = 5) and 10 female wt litter mates randomized to FLT (N = 5) or GC (N = 5), both on C57BL/6NTac background and 32 weeks of age at launch, were obtained from Taconic Biosciences (Rensselaer, NY) and designated as Experimental mice. All procedures conformed to the NRC Guide for the Care and Use of Laboratory Animals^60^ and Title 14 of the Code of Federal Regulations^61^ and approved by the NASA Institutional Animal Care and Use Committee prior to the conduct of experiments. Because the RR1 Experimental mice were flown in a study designed by Novartis investigators, animal use for this experiment was also reviewed and approved by Novartis.

**Figure 4:**
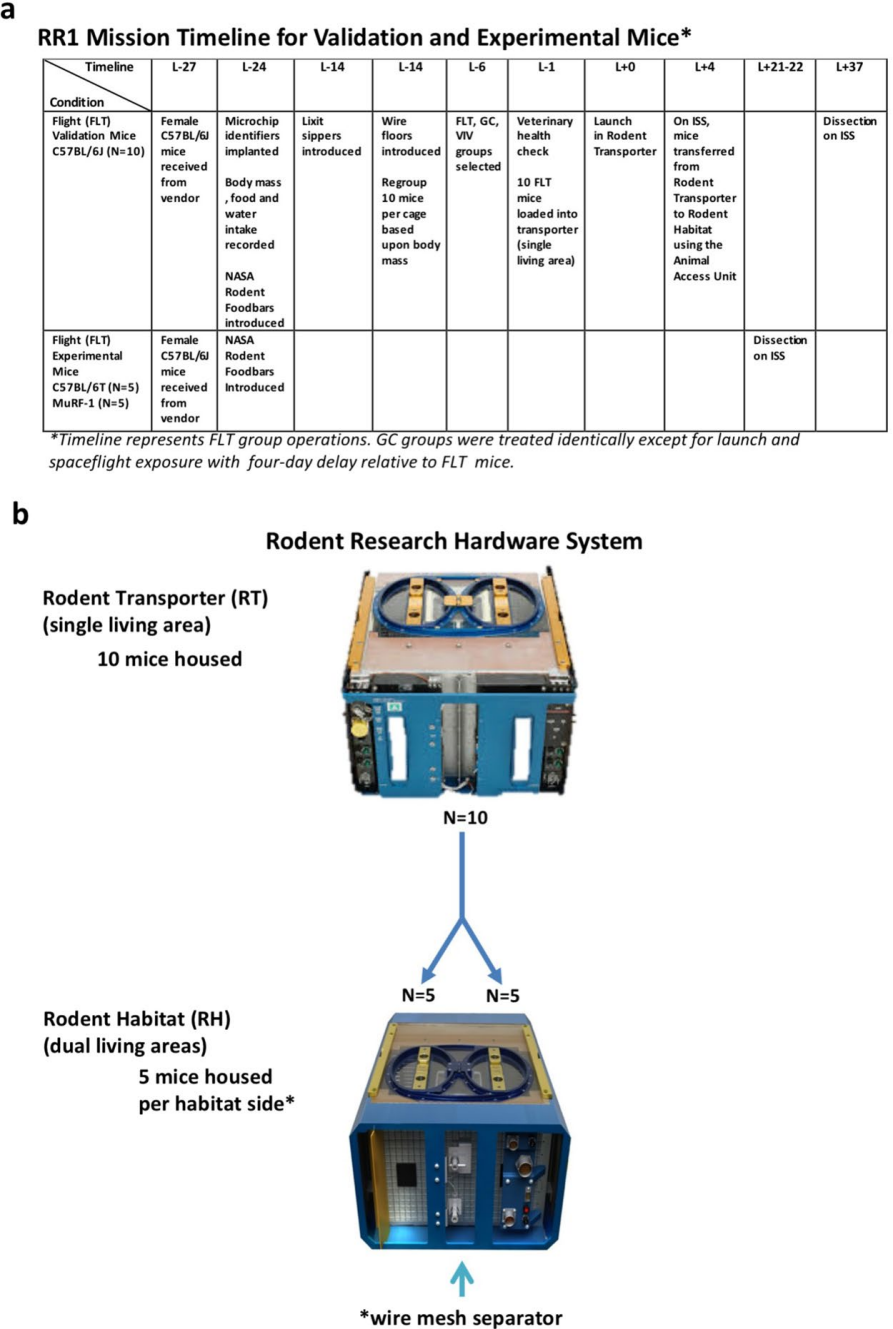
RR1 Mission Timeline and Housing. (**a**) RR1 Mission Timeline for Validation and Experimental Mice. Flight (FLT) mice treatments are shown in the timeline. Ground Control (GC) mice were treated identically with the exception of launch and exposure to spaceflight. All GC group operations were conducted for the same duration on a four-day delay relative to FLT mice. GC mice were housed in the ISS Environmental Simulator at KSC. This enabled environmental parameters (temperature, humidity, CO2) on ISS to be applied to GC mice. (**b**) The Rodent Transporter (upper image), Rodent Habitat (lower image), and Animal Access Unit (not shown) are three modules that comprise the Rodent Research Hardware System. Mice were housed in the transporter during launch and transit to the International Space Station (ISS). Following the four-day flight to ISS, the Animal Access Unit was used to provide containment during transfer of rodents between the transporter and habitat. Mice remained in the Rodent Habitat onboard the station for the duration of the mission. Photo Credits: NASA.

Timelines for the Validation and Experimental groups are shown in Fig. 4. Approximately three weeks prior to launch (L-20 to L-24), were supplied with deionized, autoclaved water via lixit spouts, and were pre-adapted to NASA Type 12 Nutrient-upgraded Rodent Food Bars (NuRFB)^62^. Food and water were available ad libitum. Mice were maintained in standard vivarium housing on a 12:12 hour dark/light cycle (lights on: 0600–1800 GMT). On L–12, mice were introduced to wire floors and regrouped into cohorts of 10. Mouse igloos were provided for enrichment from L-12 until loading into Transporters on L-1. On L-3 mice were assigned to Flight (FLT) or Ground Control (GC) conditions with groups matched for body weights. Mice assigned to a Vivarium (VIV) condition were not video-recorded thus not reported here. On L + 0 (Sept 21, 2014), ten Validation and ten Experimental FLT mice were launched on an unmanned Dragon Capsule (SpaceX4) from Kennedy Space Center (KSC) to the International Space Station (ISS) in one Transporter mouse housing unit modeled after the original NASA Animal Enclosure Module (AEM). On L + 4, mice were transferred into Rodent Habitat (RH) units on the ISS fitted with dual access ports and housing areas (Fig. 1). The RH is an advancement of the AEM used to fly rodents on numerous successful short duration (18 days or less) missions during the 30-year Space Shuttle Era. Both the AEM and RH have been shown in ground-based biocompatibility tests to adequately support rodent health for at least 90 days^63^. Transporter and RH units were each bisected by a grate separator. Mice were housed 10 per side in the Transporter unit, and five per side in the RH. GC mice were treated identically to FLT mice with the exception of launch and spaceflight. GC mice were housed in Transporter and RH units for the same duration as FLT mice, and maintained in the ISS Environmental Simulator at KSC to mirror specific environmental parameters (temperature, relative humidity, and CO_2_) on a 4-day delay relative to the FLT condition.

### Video surveillance

RH units were equipped with video capability designed to meet daily health monitor- ing requirements. Two cameras (Pinhole WD Color Model PC315XP and B/W Micro Video Model PC206XP, respectively, Supercircuits Security, Austin TX) were mounted within each habitat side, one each in close proximity to the waste filter and the lixit spouts (Fig. 1). Infra-red camera footage was acquired during the dark cycle phase and combined visible spectrum/infrared camera footage during the light cycle phase. Each RH camera feed consisted of four alternating views of the two separate cohorts using the Filter and Lixit cameras. Video programming was set to acquire one hour from the four cameras per habitat with camera feed cycling every 30 minutes for a total of 7.5 min per camera however unexpected anomalies led to schedule deviations.

Video images were acquired daily on orbit then downlinked/downloaded to the Multi-Mission Operation Center (MMOC) at Ames Research Center (ARC). Due to limited field of view, Lixit view images were excluded from behavioral analysis. Well-illuminated, higher quality Filter view images enabled detailed behavioral analysis.

### Video Sampling

RR-1 Video image acquisition procedures were devised to meet mouse health evaluation requirements, not to perform detailed behavioral analysis. For this reason, schedule, frequency, and duration of video acquisition varied daily across the mission. Videography of both Validation and Experimental mice began on L + 5. Data were analyzed from daily surveillance videos until L + 35 for Validation mice, and until L + 20 for Experimental mice. The four-day delay in experimental operations for GC relative to FLT mice enabled precise temporal features of video acquisition to be applied asynchronously to corresponding groups of GC mice at KSC. Using images acquired from left and right Filter cameras and across both phases of the dark/light cycle, behavior was sampled daily from both Validation and Experimental FLT and GC mice. A total of 6 hr video footage was acquired from Validation FLT mice and 7 hr from Experimental FLT mice and their respective GCs. Timestamps were first matched to flight logs, adjusting for intermittent loss of signal. Sample durations were precisely matched across FLT and GC cohorts, but varied across Habitat side, cycle phase, days, or age (NASA and Experimental cohorts). Video segments available for analysis from the filter view ranged from 43 to 681 seconds (Mean +/− SE: Validation, 238 +/− 168 s; Experimental, 456 +/− 272 s). Video images were acquired beginning 15 minutes to 2 hours following the start of the each 12 hr light and dark cycles. For the NASA habitat, dark cycle video was acquired on each of their 33 flight days, but light cycle video was limited to L + 5 to L + 15. For the Experimental mice, dark cycle video was acquired on each of their 17 flight days (L + 5 to L + 21, excluding L + 8 for Filter Left) and light cycle video acquired on 10 days (L + 6 to L + 20, excluding 6 flight days). Comparisons of Validation and Experimental mice were possible only until L + 20, the final video acquisition day for Experimental mice.

### Mouse behavioral analysis

Trained coders reviewed the full complement of video segments in real time and slow motion (frame-by-frame; 29.97 fps). To determine inter-rater concordance, self-grooming, allo-grooming, and feeding were independently scored by two evaluators over four or five random flight days, achieving an average inter-rater reliability of R > 0.97. Due to obvious effects of weightlessness on mice, coders were unable to score the behavior of FLT and GC mice blinded to condition. Blind scoring was performed for Experimental wild-type and MuRF1 KO mice. Tracking individual mice within and across video segments was difficult due to the absence of visible identifying markers. For behaviors observed infrequently (feeding, grooming, rearing, and social interactions), multiple mice were coded within a given video segment and these group data were expressed as ratios of behavior duration relative to the number of mice scored and video duration on the corresponding day. In contrast, activity was observed at high levels, especially during the dark phase. Ambulatory behavior was therefore analyzed in single animals across 120 second video segments.

### Around-the-clock video sampling

On L + 30, beginning at 1900 hr, twenty video samples were acquired from Validation mice hourly across a 24 hr period (video corresponding to 1700–1900 hr and 0530–0730 hr was not captured). Sample duration ranged from two to six minutes with equal sample numbers (N = 10) acquired during light and dark cycle phases. Species typical behaviors, ambulation, and circling behavior were analyzed from these segments.

### Statistical analysis

Given the inability to differentiate behavioral data derived from individual mice, housing group (N = 2 per condition) was considered to be the unit of analysis. The Student’s t-test was used to analyze data^64^ using JMP 13.1.0 (SAS Institute, Cary, NC) with the alpha level set at p < 0.05. To reduce the likelihood of Type 1 errors, Bonferroni multiple comparison corrections were used where appropriate (reducing the alpha level for some comparisons where reported in the text).

For species-typical behavior and circling of Validation mice, multiple mice were coded daily from L + 5 to L + 35 during a single video segment. Daily data were then averaged across mission halves (Fig. 2A) or mission quarters (Fig. 2B). Mission quarters were comprised of eight-day samples per average with the exception of the final quarter, where the time of video collection was outside of the sampling window, yielding only six representative samples. Daily data derived from Experimental mice were treated identically with data parsed into Q1 and Q2 bins for comparison with Validation mice that were flown on ISS for 16 days (L + 5 to L + 20). MuRF1 and wt Experimental mouse groups were combined for analysis for comparison with Validation mice (Table 1). This was justified based upon: (1) a review of the literature reporting that young MuRF1 KO and wt mice spend similar amounts of time engaged in daily wheel running^65^ and (2) similar standard deviations for behavioral measures derived from Validation and Experimental groups. For ambulation measures, a single mouse was randomly selected every other mission day. Frame-by-frame analysis was then used to quantify time spent ambulating and the morphology of limb movements.

## Supplementary information

Mice on ISS
