# Author Correction: Behavior of mice aboard the International Space Station

**DOI:** 10.1038/s41598-019-45362-1

**Published:** 2019-07-09

**Authors:** April E. Ronca, Eric L. Moyer, Yuli Talyansky, Moniece Lowe, Shreejit Padmanabhan, Sungshin Choi, Cynthia Gong, Samuel M. Cadena, Louis Stodieck, Ruth K. Globus

**Affiliations:** 1 NASA Ames Research Center, Space Biosciences Division, Moffett Field, CA 94035 USA; 2 Wake Forest School of Medicine, Obstetrics and Gynecology, Winston-Salem, NC 27101 USA; 3 Blue Marble Space Institute of Science, Seattle, WA 98154 USA; 4 Universities Space Research Association, NASA Ames Research Center, Moffett Field, CA 94035 USA; 5 San Jose State University, San Jose, CA 95192 USA; 6 KBRwyle, NASA Ames Research Center, Moffett Field, CA 94035 USA; 7 Novartis Institutes for Biomedical Research, Cambridge, MA 02139 USA; 8 BioServe Space Technologies, Department of Aerospace Engineering Sciences, University of Colorado, Boulder, CO 80302 USA; 9 Present Address: Utrecht University Graduate School of Life Sciences, Regenerative Medicine and Technology Program, Universiteitsweg 98, 3584 CG Utrecht, The Netherlands; 10 Present Address: Keck School of Medicine of the University of Southern California, Department of Molecular Microbiology and Immunology, 2011 Zonal Avenue, Los Angeles, CA 90033 USA; 11 Present Address: Duke Empirical Inc., 2829 Mission St, Santa Cruz, CA 95060 USA

Correction to: *Scientific Reports* 10.1038/s41598-019-40789-y, published online 11 April 2019

The original version of this Article contained errors. The present address for Eric L. Moyer was incorrectly given as ‘Hubrecht University Graduate School of Life Sciences, Regenerative Medicine and Technology Program, Universiteitsweg 98, 3584 CG, UTRECHT, The Netherlands’. The correct present address is listed below:

Utrecht University Graduate School of Life Sciences, Regenerative Medicine and Technology Program, Universiteitsweg 98, 3584 CG, UTRECHT, The Netherlands

In addition, in the Abstract,

“Within 7-10 days after launch, younger (but not older), mice began to exhibit distinctive circling or ‘race-tracking’ behavior that evolved into a coordinated group activity.”

now reads:

“Within 9-11 days after launch, younger (but not older), mice began to exhibit distinctive circling or ‘race-tracking’ behavior that evolved into a coordinated group activity.”

These errors have now been corrected in the PDF and HTML versions of this Article.

